# Inactivation of Cytomegalovirus in Breast Milk Using Ultraviolet-C Irradiation: Opportunities for a New Treatment Option in Breast Milk Banking

**DOI:** 10.1371/journal.pone.0161116

**Published:** 2016-08-18

**Authors:** Megan L. Lloyd, Nurul Hod, Jothsna Jayaraman, Elizabeth A. Marchant, Lukas Christen, Peter Chiang, Peter Hartmann, Geoffrey R. Shellam, Karen Simmer

**Affiliations:** 1 School of Pathology and Laboratory Medicine, The University of Western Australia, Crawley, Western Australia, Australia; 2 Marshall Centre for Infectious Diseases Research and Training, The University of Western Australia, Crawley, Western Australia, Australia; 3 School of Medical and Health Sciences, Edith Cowan University, Joondalup, Western Australia, Australia; 4 Now at the Department of Experimental Medicine, Faculty of Medicine, The University of British Columbia, Vancouver, BC, Canada; 5 Child and Family Research Institute, Vancouver, BC, Canada; 6 School of Chemistry and Biochemistry, The University of Western Australia, Crawley, Western Australia, Australia; 7 Carag AG, Baar, Switzerland; 8 School of Paediatrics and Child Health, The University of Western Australia, Crawley, Western Australia, Australia; 9 Centre for Neonatal Research and Education, The University of Western Australia, Perth, Western Australia, Australia; 10 Neonatal Clinical Care Unit, King Edward Memorial Hospital for Women, Perth, Western Australia, Australia; University of San Francisco, UNITED STATES

## Abstract

Pasteurized donor human milk is provided by milk banks to very preterm babies where their maternal supply is insufficient or unavailable. Donor milk is currently processed by Holder pasteurization, producing a microbiologically safe product but significantly reducing immunoprotective components. Ultraviolet-C (UV-C) irradiation at 254 nm is being investigated as an alternative treatment method and has been shown to preserve components such as lactoferrin, lysozyme and secretory IgA considerably better than Holder pasteurization. We describe the inactivation of cytomegalovirus, a virus commonly excreted into breast milk, using UV-C irradiation. Full replication was ablated by various treatment doses. However, evidence of viral immediate early proteins within the cells was never completely eliminated indicating that some viral gene transcription was still occurring. In conclusion, UV-C may be a safe alternative to pasteurisation for the treatment of human donor milk that preserves the bioactivity. However, our data suggests that CMV inactivation will have to be carefully evaluated for each device designed to treat breast milk using UV-C irradiation.

## Introduction

Pasteurized donor human milk (PDHM) is an important alternative to artificial formula for very preterm infants in an increasing number of neonatal intensive care units worldwide. Ideally, PDHM is provided to very preterm infants whose mothers have difficulty establishing or maintaining a breast milk supply. One of the most compelling justifications for providing PDHM instead of commercial preterm infant formula is the associated reduction in necrotizing enterocolitis (NEC), a devastating inflammatory bowel condition that is associated with extreme prematurity and can lead to bowel perforation and rapid death [1].

Donor breast milk is generally processed by Holder pasteurization (heating at 62.5°C for 30 minutes), [2] and reliably produces a microbiologically safe product reducing bacterial loads by 10^5^ colony forming units per mL [3] and effectively inactivating cytomegalovirus (CMV), a virus commonly present in breast milk that can cause severe infection in very preterm babies [4]. However, Holder pasteurization also reduces the concentration of immunoprotective proteins such as lactoferrin, lysozyme and secretory IgA, potentially moderating the beneficial effects of the breast milk [5]. Accordingly, other methods of processing breast milk that will eliminate pathogens but preserve immune factors are being assessed. Recently, Ultraviolet-C irradiation (UV-C, 254 nm) was shown to inactivate potential bacterial contaminants in breast milk (*Staphylococcus epidermidis*, *Escherichia coli*, *Enterobacter cloacae*, *Bacillus cereus*, *Staphylococcus aureus*) and to preserve lactoferrin, lysozyme and secretory IgA concentration [6,7].

While many microbial pathogens in breast milk are introduced inadvertently (e.g. skin or gut flora introduced during breast pumping), CMV will inevitably be present in many milk donations. In Australia, around 50–80% of women of fertile age are CMV seropositive prior to pregnancy [8] and the majority will experience a focal reactivation of CMV in the mammary glands with subsequent excretion of virus into breast milk for up to 2 months postpartum [9,10]. Whilst term and moderately preterm babies are generally asymptomatic after post-partum CMV infection, babies born at <28 weeks gestation are vulnerable to symptomatic infection leading to hepatitis and sepsis-like syndromes [11–13]. These babies are most likely to be offered pasteurized donor human milk, and it is important that the inactivation of CMV by UV-C is carefully validated if it is to be considered a suitable treatment for donor breast milk.

We confirm here that UV-C irradiation can effectively inactivate CMV in breast milk, but caution that an effective dose of UV-C irradiation will need to be carefully defined as new equipment is developed.

## Materials and Methods

### Cell culture

Human foreskin fibroblasts (HFF) were kindly provided by Professor Jane Allan (UWA, Perth, Australia). These were grown in Dulbecco’s Modified Enrichment Medium (DMEM), High Glucose (GIBCO), supplemented with 8% newborn bovine serum (NBS) in 5% CO_2_ at 37°C during subculture and in 2% NBS during viral titre evaluation.

### Virus stocks

A cell culture—adapted strain of CMV, AD169 [14], was kindly supplied by Professor Jane Allan (UWA, Perth, Australia) and was propagated in HFFs. High titre stock was generated by centrifugation of virus-inoculated cells (950 x g) for 30 minutes. Stocks were quantified by Tissue Culture Infectious Dose 50 analysis.

### Sample collection and ethics Statement

Frozen pasteurized breast milk was provided in 10 mL aliquots by the Hartmann Human Lactation Research Group at the University of Western Australia. Aliquots from the same stock were used for all viral inactivation studies. The use of human milk for this project was approved by the Human Research Ethics Committee of the University of Western Australia (RA/4/1/2369). All donors gave written consent for their donations to be used in research and the samples were de-identified prior to provision to our laboratory. Milk was processed by Holder pasteurization and stored in 10 mL aliquots in a -80°C freezer before use. Aliquots were thawed immediately before use.

### Tissue Culture Infectious Dose 50 (TCID_50_)

Virus stocks were diluted 10 fold in DMEM (Sigma-Aldrich, supplemented with 5% Pen/Strep (Gibco, Life Technologies) and 2% newborn bovine serum in sterile 96 well trays. 100 μL virus dilutions were inoculated onto confluent HFF cells that had been prepared in 96 well cell culture trays. Cells were incubated for 14 days (5% CO_2_, 37°C) and evaluated daily for the presence of cytopathic effect (CPE). Where characteristic cytomegalic CPE was identified, the well was assigned as “+”. The number of positive wells per tray was entered into an online calculator developed by Brett D. Lindenbach (Yale University) based on the protocol of Reed and Muench [15] to provide a value of TCID_50_/mL. All samples showing no cytopathic effect were incubated for 21 days and re-examined to ensure that there was no viral growth.

### Ultraviolet-C irradiation

The UV light box apparatus was kindly provided by the Hartmann Human Lactation Research Group (US Patent WO 2014094189, [16]). It consisted of a wooden box with a UV-C light (254 nm) attached to the top surface with a metal slide situated immediately below the light, which completely protected the sample from irradiation when closed. 180 μL (approximately 0.5 mm depth) breast milk inoculated with a ratio of 1:5 AD169 virus stock to pasteurized milk () was placed in a machine drilled well on a sterile metal sample plate (0.4 mm depth). A movable stage was used to select the appropriate distance from the light, which was measured from the top of the slide (immediately below the UV-C light) to the top of the metal well on the movable stage using a metal vernier caliper. The UV-C light was turned on for 15 minutes prior to dose measurement or sample irradiation. To deliver the dose, the sample box was placed over the prepared sample with the slide closed, the slide was opened for the appropriate amount of time, and then was closed and the light box removed. The sample was immediately processed by TCID_50_ or immediately frozen at -80°C and assessed by TCID_50_ as a batch to avoid delays in processing.

### UV-C dose measurement

The irradiation dose was measured using a Gigahertz-Optik xX911 meter with a UV-3718 detector head following the manufacturer’s methods (Gigahertz-Optik, Puchheim, Germany).

### 4°C and -20°C storage of breast milk

10 mL human milk was spiked with a ratio of 1:5 virus stock to pasteurized milk (x 10^4^. 1 mL aliquots were stored in microfuge tubes and were frozen at -20°C or stored at 4°C. At 24, 48, 72 hours, aliquots were removed, thawed (-20°C samples), diluted tenfold and assessed by TCID_50_ on previously prepared 96 well trays of confluent HFF cells. Where the same sample of infected milk was repeatedly frozen and thawed, a 10 mL sample was prepared, and frozen in a 15 mL tube (Falcon®, Corning®). This was completely thawed prior to removing 1 mL which was immediately analyzed by TCID_50_. The remaining sample was refrozen.

### Immunofluorescent staining of infected cells with a commercial monoclonal antibody

5 mL of human milk was infected with a ratio of 1:5 virus stock to pasteurized milk (). Aliquots of this mixture were treated with UV-C light (Standard 10 second time, variable 1–5 cm distance or standard 5 cm distance, variable 10–50 seconds time), and were immediately infected on confluent HFF cells on 24 well cell culture trays. Trays were centrifugally enhanced by centrifuging at 2000 x g for 30 minutes (Sigma 4K13), and were then incubated for 7 days at 37°C, 5% CO_2_. Media was removed and monolayers were washed with Tris Buffered Saline for 5 minutes. Monolayers were fixed with 1:1 Methanol: Acetone for 10 minutes at room temperature and blocked with 10% normal goat serum in TBS. Cells were washed, and incubated with 1:500 Alexa-Fluor™ 488—labelled MAB810X (Millipore) in TBS plus 10% goat serum for 30 minutes at 37°C. Cells were washed and ProLong® Gold AntiFade mountant with DAPI stain (ThermoFisher Scientific) was applied, under a coverslip and allowed to cure overnight. Cells were evaluated using a TE2000-U fluorescent microscope (Nikon, Japan). To eliminate bias, confluent microscope fields of view (10 x objective lens) were chosen on the basis of DAPI staining and were photographed using a UV-2A filter block (all cells). The same field was then immediately photographed using a B-2A block (for Alexa-Fluor™ 488 detection) to allow the proportion of fluorescently staining cells to be enumerated. Cells from both fields were automatically enumerated using ImageJ 1.48p software (NIH, USA) by either converting to a binary image (UV) or colour threshold adjustment prior to binary image conversion (B-2A) to give the percentage of infected cells (DAPI plus MAB810X double staining) per field. Particle sizes were set as a pixel size from 200 to infinity. Uninfected cells were used as a negative control, and cells infected with untreated AD169 were used as a positive control in each assay. 10 fields of view were evaluated for the variable distance evaluation and 5 fields of view were evaluated for the variable time evaluation.

### Statistics

Results are presented as mean ± standard deviation. These were calculated using GraphPad Prism 6 for Windows, Version 6.07, (GraphPad Software Inc.)

## Results

### Incubation at 4°C and -20°C does not ablate CMV viability

**Fig 1:**
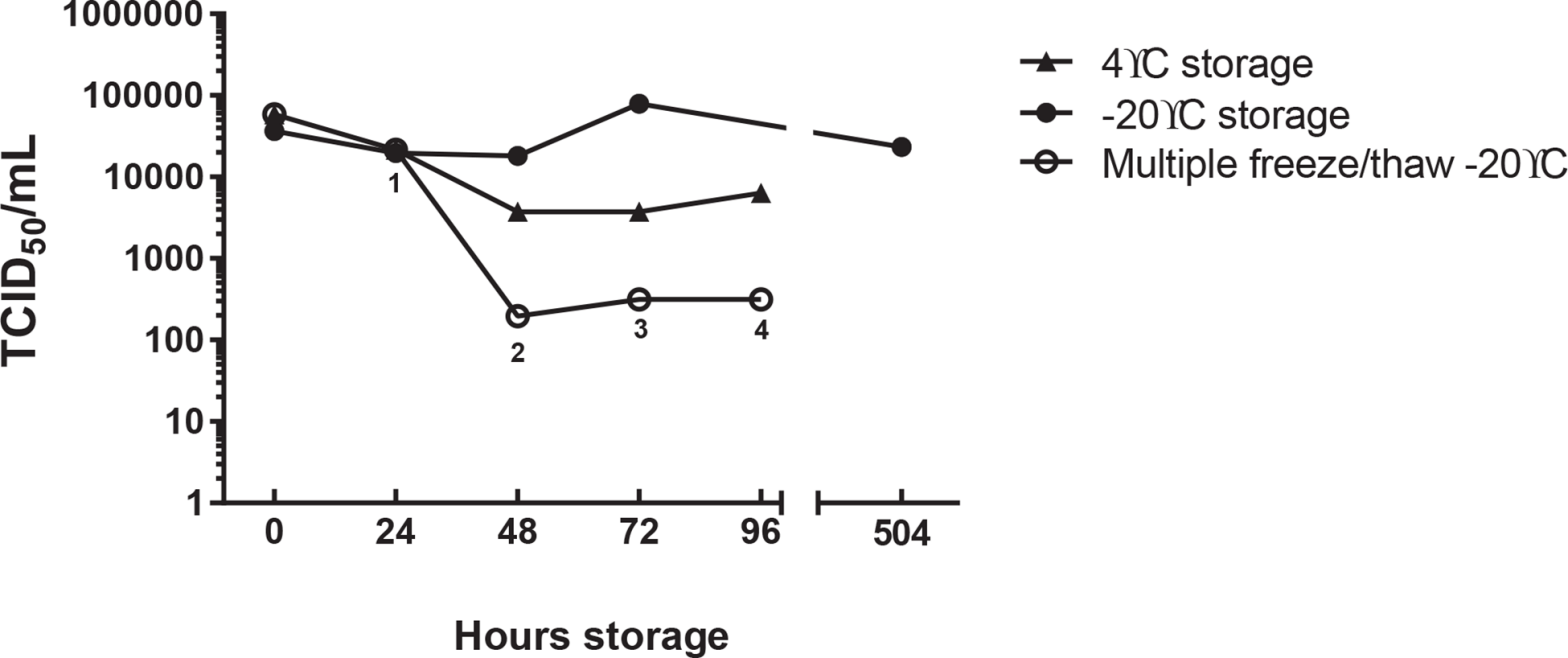
The effect of low temperatures on HCMV viability. Pasteurized breast milk was spiked with AD169 stock 1:5 and 0.5 mL aliquots were prepared and either stored at 4°C or frozen at -20°C. After 24, 48, 72 and 96 hours at 4°C or -20°C, samples were removed/thawed and analysed by TCID_50_ on human foreskin fibroblasts (HFF). One sample was stored at -20°C for 21 days (504 hours). The presence or absence of CPE was determined for each well and calculated using the Reed and Muench Calculator. Results are presented as TCID_50_/mL. Superscripts (1–4) underneath multiple freeze/thaw samples designate the number of times the sample was thawed.

Breast milk samples were inoculated with 5.9 x 10^4^ TCID_50_/ mL AD169, were stored at either 4°C or -20°C and were evaluated at daily intervals by TCID_50_. As frozen breastmilk can be safely stored significantly longer in the freezer, one sample was stored at -20°C for 21 days (504 hours). One sample stored at—20°C was repeatedly refrozen after a sample was removed. Results are presented in Fig 1 and demonstrate that viable virus was not completely inactivated after prolonged storage in these conditions.

### UV-C irradiation in milk is more effective than irradiation in media in inactivating cytomegalovirus

**Fig 2:**
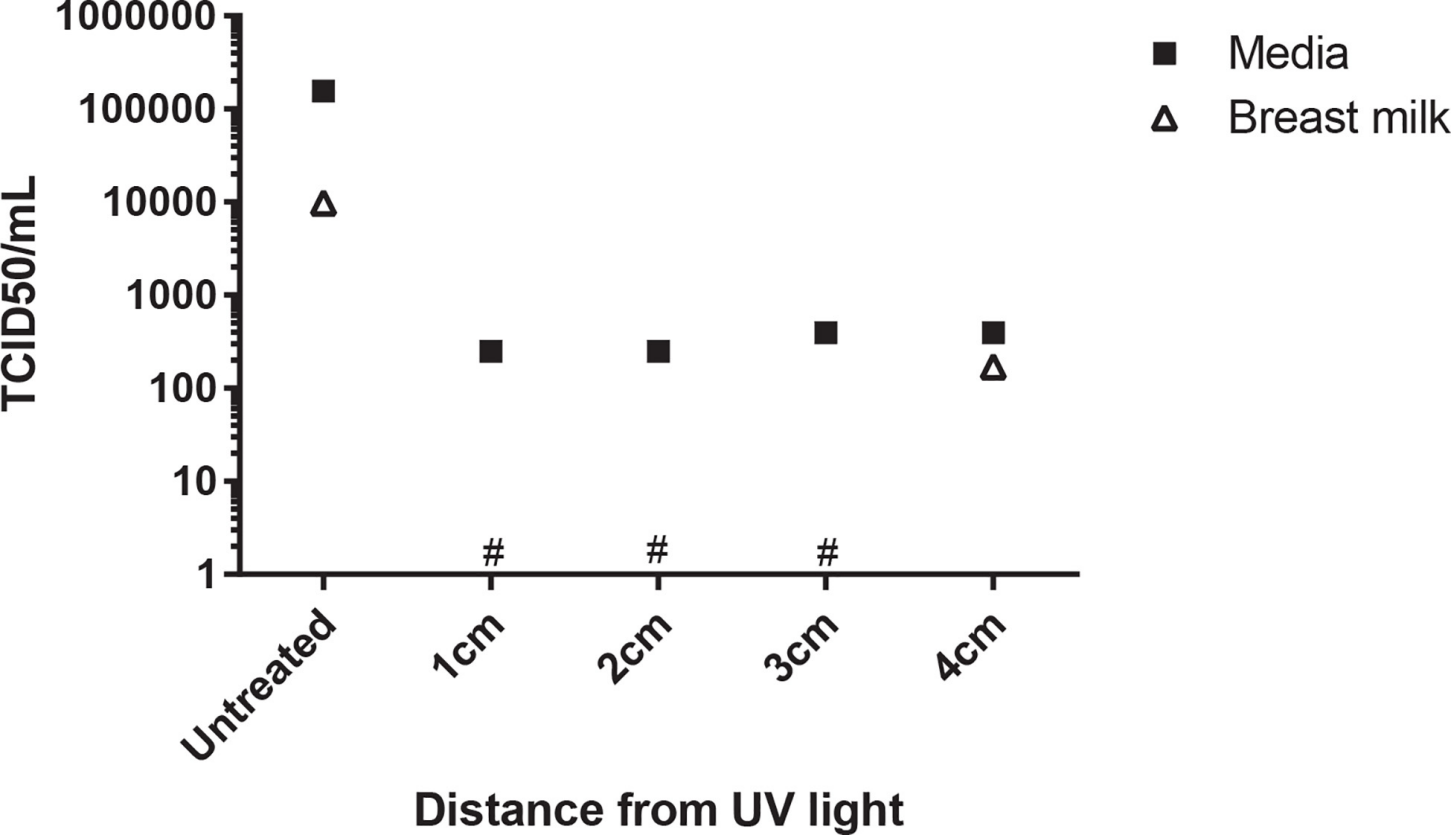
UV exposed AD169 spiked milk and media. Pasteurized breast milk or MEM culture medium were spiked with AD169 culture 1:5. 150 μL samples were irradiated for 10 seconds at 1, 2, 3 or 4 cm from the UV light source (estimated UV intensity 400, 100, 25 and 4 W/cm2 respectively). The irradiated samples were inoculated on to human foreskin fibroblasts (HFF). The presence or absence of CPE was determined for each well and calculated using the Reed and Muench Calculator. Results are presented as TCID_50_/mL. Breast milk samples with values below the limit of detection are designated (*).

Breast milk or DMEM culture media was inoculated with 1.56 x 10^5^ TCID_50_/ mL AD169 and was irradiated for 10 seconds at 254 nm at 1, 2, 3 and 4 cm from the UV light. Irradiated samples and positive (untreated) control samples were assessed by TCID_50_. UV-C irradiation for 10 seconds successfully inactivated cytomegalovirus in breast milk but minimal viability was maintained where the virus was inoculated into media (Fig 2).

### UV-C irradiation for 10 seconds successfully inactivates cytomegalovirus in breastmilk

**Fig 3:**
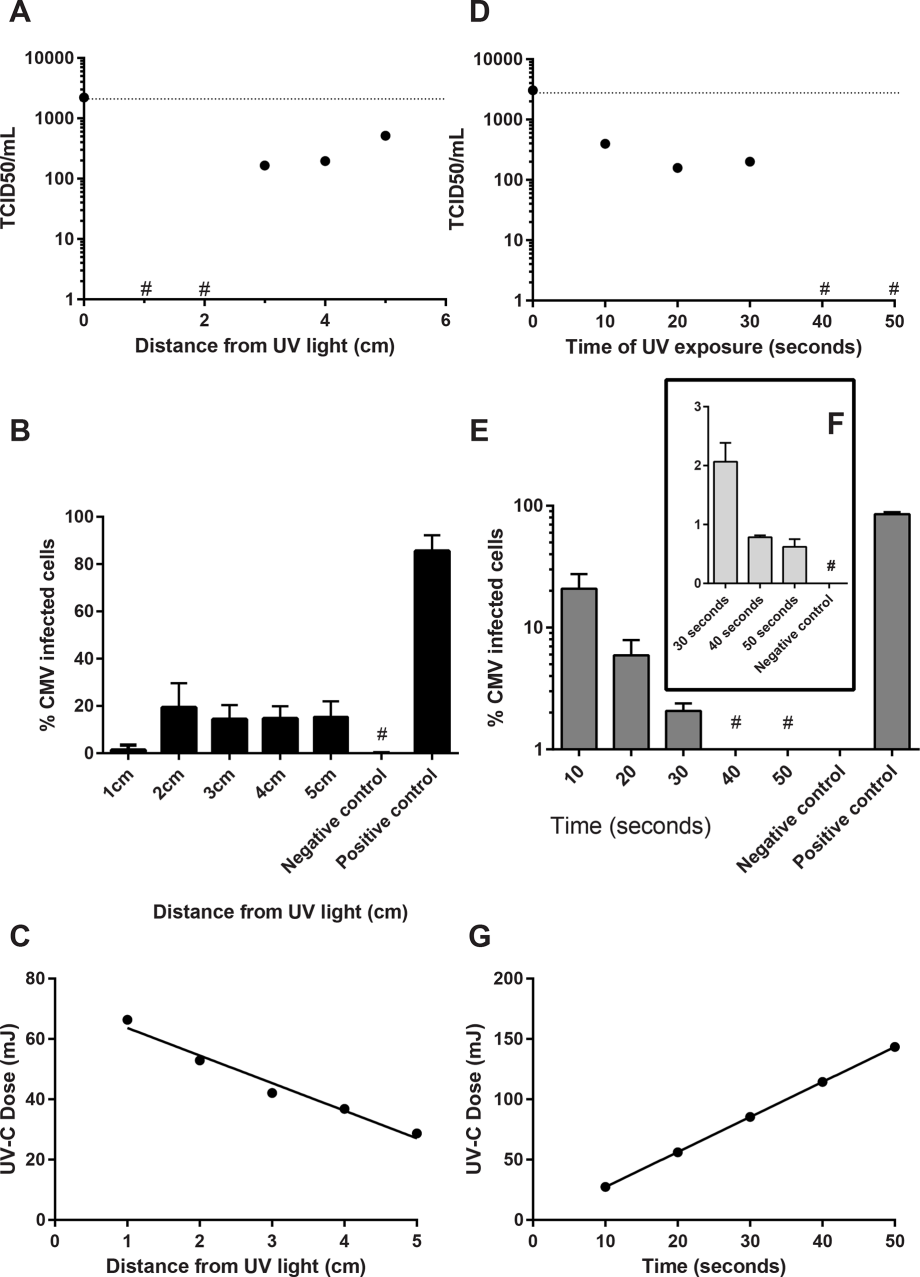
UV-C inactivation of HCMV. **A**—**C**. **UV-C exposure of spiked breast milk for 10 seconds from 1 to 5 cm from the UV light source.** Pasteurized breast milk was spiked with AD169 culture 1:5. The irradiated samples were diluted tenfold and inoculated on to human HFF and the TCID_50_ calculated using the method described by Reed and Muench [15]. **A.** Results are presented as TCID_50_/mL. Breast milk samples with values below the limit of detection are designated (*). **B.** Irradiated samples were inoculated onto HFF, centrifugally enhanced and cells were analysed by immunofluorescence at 7 days post infection. Results are presented as % infected cells. **C**: UV dose delivered. **D—F**. **UV-C exposure of spiked breast milk at 5 cm from the UV light source for different durations. D:** Pasteurized breast milk was spiked with AD169 and samples were irradiated at 5 cm from the UV light source for 10 to 50 seconds. The irradiated samples were assessed by TCID_50_. Breast milk samples with values below the limit of detection are designated (*). **E.** Irradiated samples were inoculated onto HFF, centrifugally enhanced and cells were analysed by immunofluorescence at 7 days post infection. Results are presented as % immunofluorescently labelled cells. * <1% of cells fluorescently labelled. **F**: Inset shows results for 30, 40 and 50 seconds exposure. **G**: UV dose delivered. Where error bars are present, images show mean ± standard deviation.

Breast milk inoculated with 2.2 x 10^3^ TCID_50_/ mL AD169 was irradiated for 10 seconds at 254 nm at 1, 2, 3, 4 and 5 cm from the UV light. Irradiated samples and a positive (untreated) sample were assessed by TCID_50_ (Fig 3A). An identical aliquot was treated with the same irradiation conditions and was incubated for 7 days prior to staining with MABX, which is specific for a non-structural immediate early protein. Infected cells were enumerated and are presented as the % infected cells: total cells (Fig 3B). The minimal effective UV-C dose was 53 mJ/cm^2^ (Fig 3C).

### UV-C irradiation at 5 cm successfully inactivates cytomegalovirus in breast milk

**Fig 4:**
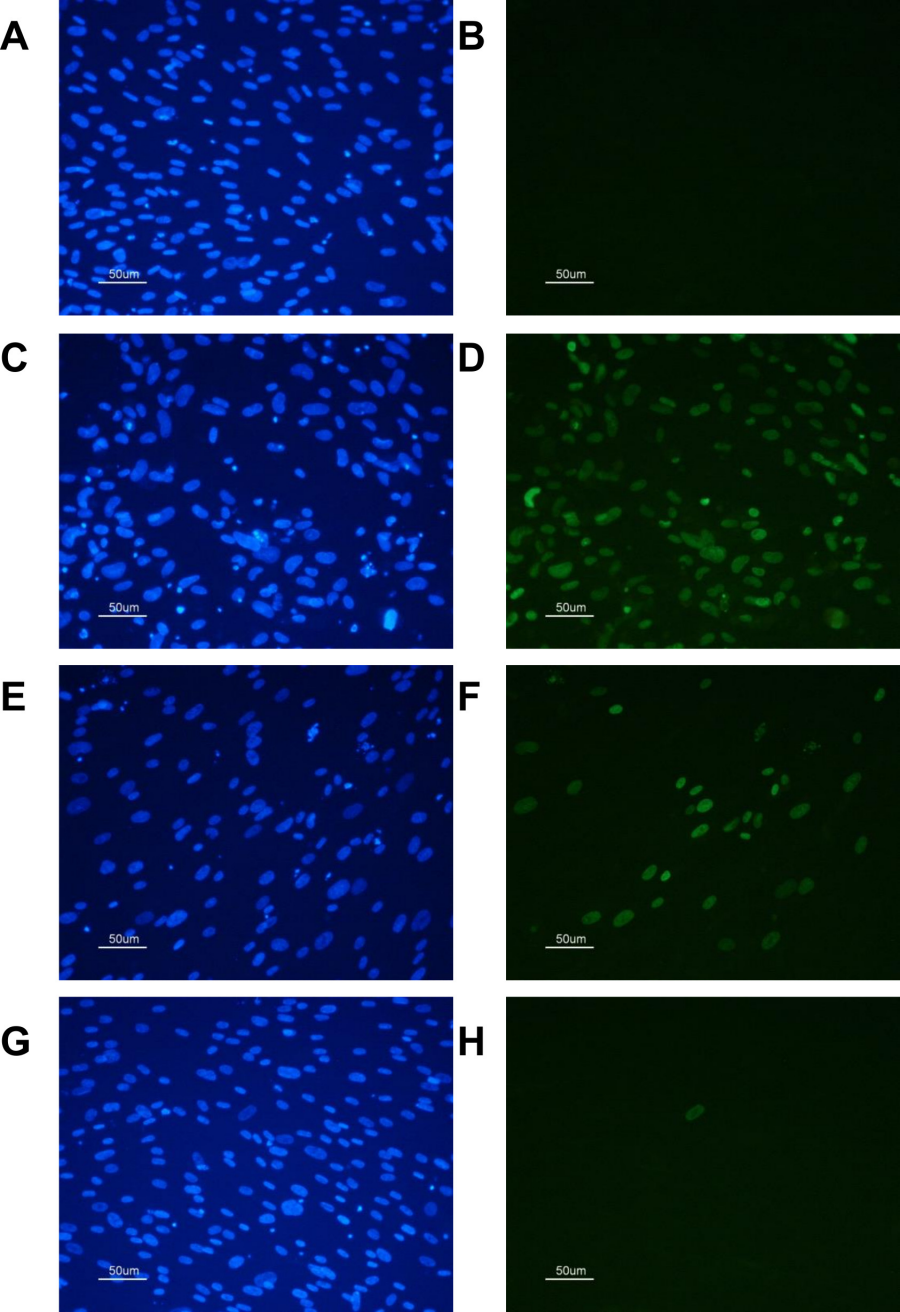
CMV infected HFF cells stained using DAPI and MABX. **A** Uninfected HFF cells (DAPI), B: Uninfected cells (MABX) **C, D:** Cells infected with AD169, no UV-C treatment, C: DAPI, D: MABX. **E, F**: Cells infected with AD169, 10 seconds UV-C treatment (5 cm), E. DAPI, F: MABX, **G, H**: Cells infected with AD169, 50 seconds UV-C treatment (5 cm) G: DAPI, H: MABX.

Breast milk inoculated with 3.1 x 10^3^ TCID_50_/ mL AD169 was irradiated at 5 cm from the UV light for 10, 20, 30, 40 and 50 seconds. Irradiated samples and a positive (untreated) sample were assessed by TCID_50_ (Fig 3D). An identical aliquot was treated with the same irradiation conditions and was incubated for 7 days prior to staining with MABX. Infected cells were enumerated and are presented as the % infected cells: total cells. Results are presented in Fig 3E. At 30 seconds exposure (85 mJ/cm^2^), 2% of cells were immunofluorescent. At 40 seconds exposure (114 mJ/cm^2^), less than 1% cells were immunofluorescent. This result was not improved by 50 seconds irradiation (143 mJ/cm^2^, Fig 3F inset). The UV dose was measured as previously described (Fig 3G). Fig 4 shows an example of the typical staining achieved using DAPI and MABX.

## Discussion

UV-C irradiation (200–280 nm) is currently used extensively to eliminate microbial contamination in the food industry. We are currently investigating the ability of 254 nm UV-C, which has previously been shown to reduce bacterial viability while simultaneously retaining the biological activity of the milk [6,7], for its ability to inactivate cytomegalovirus.

Both optimal (1cm) and suboptimal (5cm) UV-C irradiation protocols were evaluated with the latter demonstrating efficacy where prolonged irradiation was provided. Irradiation of CMV infected breast milk with 64 mJ/cm^2^ UV-C was shown to eliminate replicative virus and to reduce the detection of intracellular CMV protein to less than 1% of cells in culture. Although no replication competence was demonstrated (defined by the absence of CPE, Fig 3A), residual intracellular viral proteins were detected using a monoclonal antibody. UV-C irradiation is known to affect cell viability by inducing DNA damage such as cyclobutane pyrimidine dimers that impede DNA replication [17]. MABX is specific for a non-structural immediate early protein, and this can theoretically be transcribed without complete replication and virion production occurring. Limited immediate early transcription in CMV latency has previously been demonstrated *in vitro* in mouse lung tissue after infection with murine cytomegalovirus and no translation of early or late proteins was detected [18]. The residual cells demonstrating fluorescent staining after prolonged irradiation (40 and 50 seconds, 140 and 180 mJ/cm^2^ respectively) may therefore not be indicative of future productive infection. Evidence for this was provided by the prolonged incubation (up to three weeks) of identically treated virus in 96 well trays (for TCID_50_) that did not produce plaques (AD169 is a cell culture-adapted virus and produces identifiable CPE within 7 days). To further evaluate replication competence, future studies will assess the transcription of late CMV genes after UV-C treatment. This will be important as we cannot completely exclude the possibility that viral replication is occurring.

Some natural suppression of CMV replication is suggested by the differing effects of CMV inoculated into media (less effective inactivation at all treatment distances) than pasteurized breast milk (Fig 2). All virus detected was at the limit of detection, however the result was consistent and demonstrates that cell culture media is not an appropriate substitute for breast milk when evaluating UV-C irradiation. Interestingly, UV-C would be expected to penetrate culture media with greater efficiency than the more opaque breast milk. One of the obstacles with using this technology to treat liquids such as milk is the difficulty with radiation penetrance (estimated to be 0.5mm), and new methodologies will need to be developed to allow larger volumes of milk to be treated [19].

The ingestion of breast milk containing viable CMV can result in postpartum infection in very preterm babies [9]. The severity of infection is varied and a detrimental effect on the long term developmental outcomes for infected infants has not consistently been shown. Illustrating the confusion surrounding this area, while some investigators have concluded that post-partum CMV infection is a relatively mild and self-limiting infection with no obvious long term sequelae [20], others have published case studies of infants that are severely affected by infection [12,21] and a recent multi-centre study demonstrated an association between postnatal CMV infection and increased risk of bronchopulmonary dysplasia [22]. In 2010 an evaluation of the current post-partum CMV infection literature concluded that no statements could be justified that either endorsed or did not recommend treatment of breast milk where mothers were excreting CMV [23]. Very few long term follow up studies have been carried out and are hampered by small numbers of participants producing underpowered studies [24,25]. A recent report describing intellectual impairment in adolescents suggests that long term sequelae are measureable [26].

Many neonatal intensive care units currently freeze breast milk at -20°C for up to 3 days prior to feeding to very preterm infants to reduce CMV titres. The efficacy of this treatment has been controversial with some investigators showing a significant reduction in viral titre and some showing little benefit (discussed in [27]) including a recent study that reported a randomized trial of milk freezing that did not show a reduction in CMV infections [28]. We did not find a reduction in CMV viability associated with storage at -20°C and saw little effect on viability after 21 days (504 hours) storage. Whilst these experiments were carried out using the cell culture-adapted strain of CMV, AD169, these data may illustrate the worst case scenario at the peak of CMV excretion into breast milk (illustrated in [29]). Certainly, other investigators have concluded that freezing breast milk does not ultimately protect susceptible infants from infection [30]. It is possible that a low titre of CMV excreted into breast milk is inactivated by freezing, but that this effect is overwhelmed as more virus is excreted as lactation proceeds [31]. Prior CMV infection should not be a contraindication for breast milk donation or for breast feeding generally. However, the risk to the population being provided with donor breast milk (generally very preterm infants <32 weeks gestational age) is significant, and this potential pathogen is eliminated by Holder pasteurization.

Reports of pathology associated with post-partum infection with CMV in infants born at >28 weeks gestational age are relatively rare, and it is possible that severely symptomatic infections and adverse long term outcomes are sometimes diluted by age categories that are too broad. As the survival of infants born at 23 to 25 weeks gestational age improves worldwide, the long term sequelae of postpartum CMV infection may become more evident. Holder pasteurization of breast milk from CMV seropositive mothers is performed in some neonatal facilities. However, a recent report stemming from a transition from pasteurization to providing untreated expressed breast milk in an Austrian neonatal unit demonstrated a non-significant reduction in NEC and an increase in CMV infections with no increase in mortality suggesting that the benefits of routine Holder pasteurization for this population are minimal and may even cause harm [32]. An alternative method of milk treatment that effectively inactivates CMV and simultaneously maintains other immunoprotective elements may reduce infant disease in the neonatal intensive care unit.

UV-C treated human milk retains substantial bioactivity while effectively killing bacteria and inactivating the replicative capacity of CMV in breast milk. Other potential pathogens that are also excreted into breast milk such as Human Immunodeficiency Virus will need to be evaluated before this alternative treatment is approved for use in the context of a human milk bank.
